# Beyond static snapshots: predicting dynamic, explainable intermediate phenotypes for climate-resilient crop breeding

**DOI:** 10.3389/fpls.2026.1873747

**Published:** 2026-06-30

**Authors:** Shan Jiang, Jun Yan

**Affiliations:** 1 National Nanfan Research Institute, Chinese Academy of Agricultural Sciences, Sanya, Hainan, China; 2 State Key Laboratory of Maize Bio-breeding, National Maize Improvement Center, Frontiers Science Center for Molecular Design Breeding, College of Agronomy and Biotechnology, China Agricultural University, Beijing, China

**Keywords:** crop stress resilience, dynamic intermediate phenotypes, genomic prediction, high-throughput phenotyping (HTP), multi-omics integration

## Introduction

The convergence of multi-omics profiling, high-throughput phenotyping (HTP), and artificial intelligence (AI) has expanded our ability to characterize crop stress responses at unprecedented resolution (Danilevicz et al., 2025; Pacheco-Ruiz et al., 2025; Tsega and Mullualem, 2026). Researchers can now routinely identify candidate genes, construct gene regulatory networks, and train machine learning models to predict terminal phenotypes, such as yield under drought, biomass under salinity, and disease severity scores. Yet the practical impact remains limited: delivering a single improved crop variety to market still requires approximately one decade and more than 14 million euros, a timeline that has barely changed in 30 years despite the exponential growth in data generation (Pacheco-Ruiz et al., 2025). We argue that a fundamental cause of this translational gap is what we call the *temporal poverty* of current GP: models often predict static endpoints rather than the dynamic processes that determine them.

Classical GP models, including multi-omics-informed variants, are overwhelmingly trained on traits measured at single time points, such as final yield or end-of-season stress scores. However, stress tolerance is inherently temporal, reflecting a cascade of physiological decisions whose sequence and timing ultimately determine field survival: when to close stomata, how rapidly to accumulate osmolytes, and whether to prioritize root extension or shoot preservation. A model that predicts terminal drought tolerance without distinguishing whether it arises from early water conservation, sustained photosynthesis, or post-stress recovery offers limited support for rational gene stacking or knowledge transfer across environments and crops (Danilevicz et al., 2025). Based on these observations, we hypothesize that making the dynamic trajectories of intermediate physiological traits the direct targets of GP, rather than treating them as auxiliary variables, will substantially improve prediction accuracy, mechanistic interpretability, and the rate of genetic gain for stress resilience in crop breeding.

**Figure 1:**
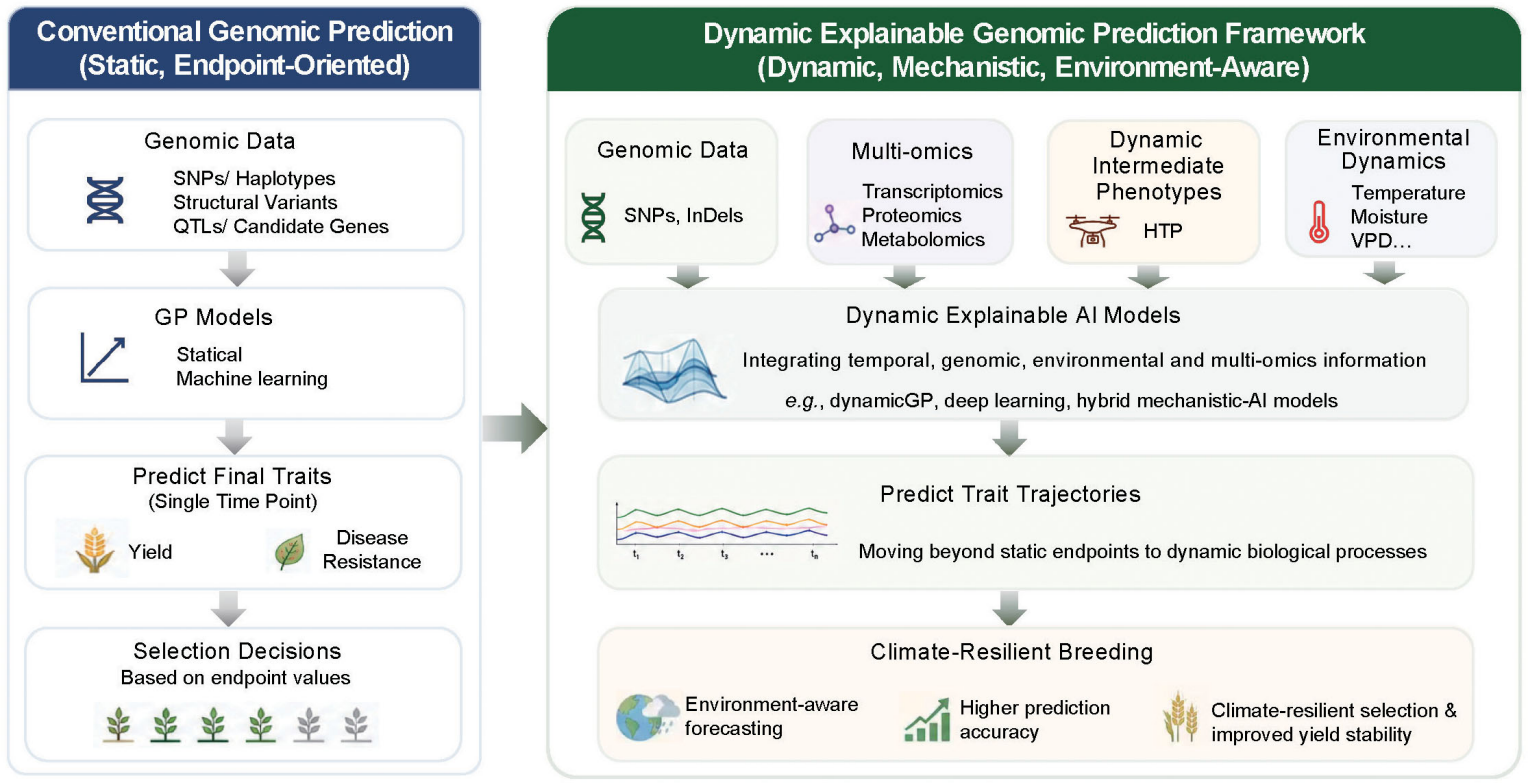
Bridging the temporal gap in genomic prediction. Conventional genomic prediction focuses on static endpoint prediction, whereas the proposed framework targets dynamic intermediate phenotypes and integrates environmental and multi-omics information to enable trajectory-aware, interpretable prediction for climate-resilient crop breeding.

To bridge this temporal gap, we propose a dynamic explainable genomic prediction framework centered on intermediate physiological trajectories (**Figure 1**). The framework integrates dynamic phenotyping, environmental information, and mechanistic multi-omics anchors to support trajectory-aware prediction and climate-resilient breeding.

## From static endpoints to dynamic trajectories: recent computational advances

This reframing is becoming increasingly feasible. The recent dynamicGP model combines GP with dynamic mode decomposition (DMD) to predict full temporal trajectories of multiple morphometric and colorimetric traits scored by HTP in maize and *Arabidopsis thaliana* (Hobby et al., 2025). DynamicGP was also shown to predict traits at time points beyond the training period. It was the only model tested with this capability and showed superior longitudinal accuracy in capturing developmental dynamics (Hobby et al., 2026). The model further revealed that traits with more temporally stable heritability can be predicted with higher accuracy, providing practical guidance on which dynamic intermediates to prioritize (Hobby et al., 2025). Mechanistic modeling offers a complementary line of evidence: computational models of grass inflorescence morphodynamics recently guided the discovery of the *duo2* mutant allele in wheat, which accelerates developmental progression and improved yield by 7-11% under field conditions (Wang et al., 2026b). These two independent advances, one data-driven and one mechanism-driven, converge on the same insight: incorporating time as a central dimension in prediction can provide predictive power and mechanistic understanding that are inaccessible to static models, thereby connecting prediction with actionable breeding decisions.

## An asymmetric integration strategy: HTP as temporal skeleton, multi-omics as mechanistic anchors

A realistic view of current data availability shapes this strategy. The current and near-future foundation for dynamic intermediate phenotype prediction is largely HTP. The marginal cost of repeated phenotyping with automated platforms, UAVs, and low-cost sensors has decreased substantially, and public time-series datasets covering wheat, sorghum (LeBauer et al., 2020), soybean, and several other crops are rapidly expanding. This supports a pragmatic, asymmetric integration strategy: HTP provides the dense temporal skeleton of dynamic trait trajectories, while multi-omics is deployed selectively at a small number of mechanistically critical time windows, such as the onset of stress signaling, the transition from alarm to acclimation, or the peak of a known physiological trade-off. These sparse but information-rich omics snapshots serve as “explainability anchors.” When explainable AI approaches are used to link these molecular profiles to parameterized dynamic modes extracted from HTP, the resulting model can reveal which genes, transcripts, or metabolites modulate specific phases of the dynamic response and when. For instance, SHAP-based interpretation of Random Forest models applied to soybean multi-omics data revealed that the isoflavone derivative daidzin and specific drought-tolerant microbes are major contributors to phenotypic variation under drought stress, while SHAP-based interaction networks uncovered cross-omics links between metabolites and microbial taxa (Yoshioka et al., 2026). Extracting biologically meaningful signals from such comparisons requires specialized computational tools. The MODAS2 pipeline, for example, uses contrastive principal component analysis, a machine learning algorithm, to disentangle stress-responsive molecular QTLs from background genetic effects in multi-omics data, enabling the identification of salt-responsive genetic variants in maize (Liu et al., 2025). Such tools are essential for implementing the explainability-anchor strategy at the stress-transition windows targeted by our framework.

## Complementary roles in the breeding pipeline and pathways to genetic gain

Advocating for dynamic intermediate phenotypes as prediction targets does not mean abandoning terminal agronomic traits. The two approaches serve complementary roles at different stages of the breeding pipeline. In early-generation selection, when thousands of lines must be evaluated and resources for multi-environment yield trials are limited, dynamic intermediates, which are often measurable earlier, at lower cost per data point, and with higher heritability, can help enrich populations for stress-resilient candidates (Melsen et al., 2025). In later-stage trials, direct prediction of yield under target stress environments remains indispensable for final variety release decisions. In practice, the central question is not which target is superior in isolation, but whether their combined use can accelerate genetic gain for stress tolerance.

Dynamic intermediate phenotypes can contribute to genetic gain through three main routes. First, many dynamic intermediates show higher heritability and earlier measurability than terminal yield under stress, enabling more accurate early-generation selection and shorter breeding cycles. Second, a dynamic physiological module, such as rapid osmotic adjustment within 48 hours of soil drying, can become a reusable building block once it has been genetically dissected and validated. Such modules could then be stacked, introgressed, or transferred across genetic backgrounds and crop species. Third, selecting on the shape of a response curve rather than a single terminal value can reduce environmental noise, because temporal patterns are often more genetically determined than absolute end-point values (Hobby et al., 2025). This framework is also inherently cross-crop: a conserved physiological module such as “stomatal response speed to soil drying” is unlikely to be restricted to a single species. Orthologous genes and conserved pathways can inform candidate selection in legumes, vegetables, and under-researched crops, extending advanced breeding methodologies beyond the major cereals (Kundu and Tanti, 2026; Wang et al., 2026b).

## Incorporating environmental dynamics into prediction models

The dynamic GP framework we advocate remains incomplete without explicit environmental inputs. At present, many AI-driven prediction models treat the environment as a categorical label or a set of static summary statistics. This approach can conflate genetic and environmental effects and limit performance forecasting across mega-environments or novel climatic scenarios. To support the development of climate-resilient varieties, models should instead incorporate environmental data as an explicit, dynamic data layer, including time series of temperature, soil moisture, and vapor pressure deficit, that co-determines the trajectory of intermediate phenotypes. Recent advances suggest that this is increasingly feasible. Enviromics and reaction-norm approaches that incorporate high-dimensional environmental covariates through penalized regression can now approach the accuracy of deep learning while retaining interpretability and an explicit description of genotype-by-environment interactions (Avagyan et al., 2025). In parallel, hybrid frameworks that couple crop growth models with whole-genome prediction can link genetic, environmental, and management inputs to dynamic physiological outputs (Laurent et al., 2025). Building on these developments, embedding environmental time series into the temporal kernels of dynamic models such as dynamicGP could, in principle, enable prospective, environment-aware forecasting of genotype-specific response curves. This would shift selection from retrospective mega-environment classification toward forward-looking prediction across single or multiple target mega-environments. However, robust extrapolation to untested environments and stress combinations remains a key open challenge. Careful envirotyping and cross-environment validation will therefore be essential before such models can guide variety deployment decisions in practice.

## Discussion

The framework proposed here has important implications for future crop production. By shifting the breeding target from terminal yield to the temporal architecture of stress responses, breeders could develop selection strategies that are both physiologically informed and operationally efficient. Dynamic intermediate phenotypes, captured through increasingly affordable HTP platforms, can serve as early indicators of resilience, allowing breeders to discard susceptible lines long before harvest. This could shorten the breeding cycle, especially when combined with genomic selection and speed breeding, while also enabling the deliberate assembly of stress-resilience modules that are robust across environments. Empirical evidence already shows that integrating HTP-derived spectral data with genomic information can substantially improve cross-environment prediction accuracy (McBreen et al., 2025; Nannuru et al., 2025). As climate variability intensifies, the ability to design varieties with predictable temporal behavior under drought, heat, or salinity will become increasingly important for enhancing yield stability and food security.

Challenges remain. Dynamic GP models have yet to be systematically validated across radically different environments, and their ability to predict trait dynamics under novel stress combinations remains unproven. Data from controlled HTP platforms must also be calibrated against field conditions. In addition, the integration of heterogeneous multi-omics datasets creates persistent bottlenecks that constrain practical application beyond proof-of-concept studies (Pacheco-Ruiz et al., 2025; Tsega and Mullualem, 2026). Multi-omics time series, even at the sparse sampling density we advocate, remain limited by high technology costs and scalability challenges that restrict their routine deployment in breeding programs (Syeda, 2025; Younas et al., 2025). However, pilot-scale time-series multi-omics studies demonstrate both feasibility and value. For example, transcriptomic and ionomic profiling of *Sorghum bicolor* across a 21-day micronutrient stress time course revealed iron-zinc regulatory crosstalk and conserved gene regulatory networks (Mishra et al., 2025), while high-resolution time-series transcriptomic and metabolomic profiling of salt-tolerant and salt-sensitive maize inbred lines identified the hub gene *ZmGLN2* and constructed dynamic regulatory networks governing salt-responsive metabolite biosynthesis (Zhang et al., 2025). Furthermore, the MODAS2 pipeline, which uses contrastive principal component analysis to extract stress-responsive signals from multi-omics comparisons, illustrates that the computational methods needed to integrate heterogeneous omics layers are already under active development (Liu et al., 2025).

Equally important is the ability of AI models to capture the non-linear genetic interactions that underpin complex stress responses. Linear mixed models, the backbone of classical GP, primarily model additive effects and therefore have limited capacity to capture epistasis, gene-by-environment interactions, and threshold-type responses (Wang et al., 2026a). In contrast, deep learning architectures can learn hierarchical, non-linear mappings from high-dimensional input spaces. When applied to dynamic intermediate phenotypes, these models could learn not only which genomic regions influence the temporal shape of a trait, but also how those regions interact with each other and with environmental triggers over time. For example, convolutional neural networks have outperformed classical methods such as LASSO and Bayes C in predicting integrative traits, with the combination of CNNs and crop model parameters further enhancing prediction accuracy (Larue et al., 2024). Fully harnessing non-linear interactions for dynamic trait prediction will likely require hybrid approaches that embed mechanistic constraints into flexible AI architectures, ensuring that predictions remain both powerful and biologically plausible.

As outlined in the preceding section, integrating environmental time series into dynamic GP models remains a frontier, but the computational tools and conceptual frameworks are now within reach. The integration of environmental data with multiple omics layers for genotype-by-environment prediction has been identified as a major emerging frontier, although it is currently addressed in fewer than 20% of studies (Tsega and Mullualem, 2026). This gap underscores the urgency of the framework we advocate. These are not sequential prerequisites, but parallel investments that reinforce one another. We therefore call on the crop science community to: (i) prioritize the generation of time-series phenotypic data in multi-environment stress trials, leveraging increasingly affordable HTP platforms; (ii) adopt explainable AI as standard practice, not merely reporting prediction accuracy but also elucidating which features drive predictions, when, and through which physiological mechanisms (Danilevicz et al., 2025); and (iii) develop selection indices that explicitly reward favorable dynamic trajectories alongside terminal trait values. Multi-omics and AI should not merely describe how stress resistance appears at harvest; they must reveal how it unfolds over time and how it can be rationally assembled. Bridging this temporal gap will connect current data abundance with the practical goal of delivering climate-resilient crops to farmers’ fields.

In conclusion, the temporal poverty of current genomic prediction represents a fundamental bottleneck in translating multi-omics and AI advances into climate-resilient crops. By placing dynamic, explainable intermediate phenotypes at the center of prediction and selection, we can move beyond correlative rankings toward a mechanistic understanding of stress resilience that is both actionable and transferable. The computational tools, phenotyping infrastructure, and pilot datasets are already available; what is now required is a community-wide commitment to time-aware breeding strategies. Bridging this temporal gap will help connect current data abundance with the practical goal of delivering stable, high-performing varieties to farmers’ fields under increasingly unpredictable climatic conditions.
